# Editorial: From theory to practice: the latest developments in neuromorphic computing applications

**DOI:** 10.3389/fnins.2024.1511987

**Published:** 2024-10-30

**Authors:** Arash Ahmadi, Shaghayegh Gomar, Majid Ahmadi

**Affiliations:** ^1^Department of Electronics, Carleton University, Ottawa, ON, Canada; ^2^Department of Systems and Computer Engineering, Carleton University, Ottawa, ON, Canada; ^3^Department of Electrical and Computer Engineering, University of Windsor, Windsor, ON, Canada

**Keywords:** neuromorphic, spiking neural network, neuromorphic computing, neuromorphic engineering, bio-inspired computing

The field of artificial intelligence has witnessed remarkable progress in recent years, with Artificial Neural Networks (ANNs) at the forefront of this revolution. These conceptual models of biological neurons have demonstrated unprecedented capabilities in tackling complex problems such as classification, pattern recognition, and forecasting. However, as we push the boundaries of ANN applications, we face a significant challenge: the high computational demands and associated energy consumption of these models, particularly in large-scale problems and resource-limited applications.

Enter neuromorphic engineering (NE) and neuromorphic computing (NC), two closely related fields that offer promising solutions to these pressing issues. NE aims to replicate the behavior of biological neural networks in circuits and systems, while NC focuses on developing applications based on these bio-inspired principles. These interdisciplinary areas have attracted researchers from diverse backgrounds, including neuroscience, physics, computer science, electrical engineering, and computer engineering, fostering a rich ecosystem of ideas and innovations.

In recent years, we have seen significant advancements in the implementation of bio-inspired neuron and network models using digital, analog, and mixed-signal circuits and systems. These developments have brought us closer to replicating brain functionality in artificial systems. However, the full potential of neuromorphic computing remains to be realized, and there is still much to explore in terms of practical applications and real-world implementations.

This Research Topic, “*From theory to practice: the latest developments in neuromorphic computing applications*,” aims to bridge the gap between theoretical advancements and practical applications in the field of neuromorphic computing. We sought contributions that showcase the latest developments in neuromorphic hardware, novel architectures, and innovative applications across various domains.

We are pleased to present a Research Topic of cutting-edge research articles that contribute significantly to this rapidly evolving field:

1. SHIP: a computational framework for simulating and validating novel technologies in hardware spiking neural networks by Gemo et al. introduces a powerful computational framework for simulating and validating new technologies in hardware spiking neural networks. Spiking Hardware In the loop Platform (SHIP) provides researchers with a valuable tool for exploring and optimizing neuromorphic designs before physical implementation. This framework bridges the gap between software simulations and hardware prototyping, potentially accelerating the development of neuromorphic systems.
2. A comprehensive review of advanced trends: from artificial synapses to neuromorphic systems with consideration of non-ideal effects by Kim et al. offers an in-depth analysis of the latest trends in neuromorphic computing, focusing on the journey from artificial synapses to complete neuromorphic systems. This review is particularly valuable as it considers non-ideal effects, providing a realistic perspective on the challenges and opportunities in the field. By highlighting advanced trends, this work serves as an excellent resource for researchers and practitioners aiming to push the boundaries of neuromorphic technology.
3. The silence of the neurons: an application to enhance performance and energy efficiency by Heidarpur et al. presents an innovative approach to improving the performance and energy efficiency of neuromorphic systems. By leveraging the concept of neuronal silence, the authors demonstrate how to optimize the operation of spiking neural networks. This work addresses one of the key challenges in the field—energy efficiency—and offers a promising direction for future research and development in neuromorphic computing.
4. Real-time execution of SNN models with synaptic plasticity for handwritten digit recognition on SIMD hardware by Vallejo-Mancero et al. showcases a practical application of neuromorphic computing in the domain of handwritten digit recognition. The authors demonstrate the real-time execution of Spiking Neural Network (SNN) models with synaptic plasticity on Single Instruction, Multiple Data (SIMD) hardware. This work illustrates the potential of neuromorphic approaches in real-world pattern recognition tasks and highlights the importance of hardware-software co-design in achieving efficient neuromorphic systems.

These contributions collectively demonstrate the diverse applications and potential of neuromorphic computing across various domains. From novel simulation frameworks and comprehensive reviews to innovative approaches for enhancing efficiency and practical applications in pattern recognition, the articles in this Research Topic showcase the breadth and depth of current research in the field.

As we move forward, it is clear that neuromorphic computing has the potential to revolutionize many aspects of our technological landscape. By mimicking the efficiency and adaptability of biological neural systems, neuromorphic approaches offer promising solutions to the energy consumption and scalability challenges faced by traditional computing paradigms. Moreover, the unique properties of neuromorphic systems, such as inherent parallelism and event-driven processing, open up new possibilities for real-time, low-power applications in areas like edge computing, Internet of Things (IoT), and autonomous systems.

However, challenges remain. The field must continue to address issues such as scalability, reliability, and integration with existing technologies. Additionally, there is a need for standardized benchmarks and evaluation metrics to facilitate fair comparisons between different neuromorphic approaches and traditional computing methods.

As guest editors, we are excited by the progress showcased in this Research Topic and the future directions it suggests. We believe that continued collaboration between researchers from diverse backgrounds will be crucial in driving the field forward and translating theoretical advances into practical, real-world applications.

We invite readers to explore the articles in this Research Topic and hope that they will serve as a source of inspiration for future research and development in neuromorphic computing. As we stand at the intersection of neuroscience, engineering, and computer science, the potential for groundbreaking innovations in neuromorphic systems has never been greater. We look forward to witnessing the continued evolution of this fascinating field and its impact on the future of computing.

